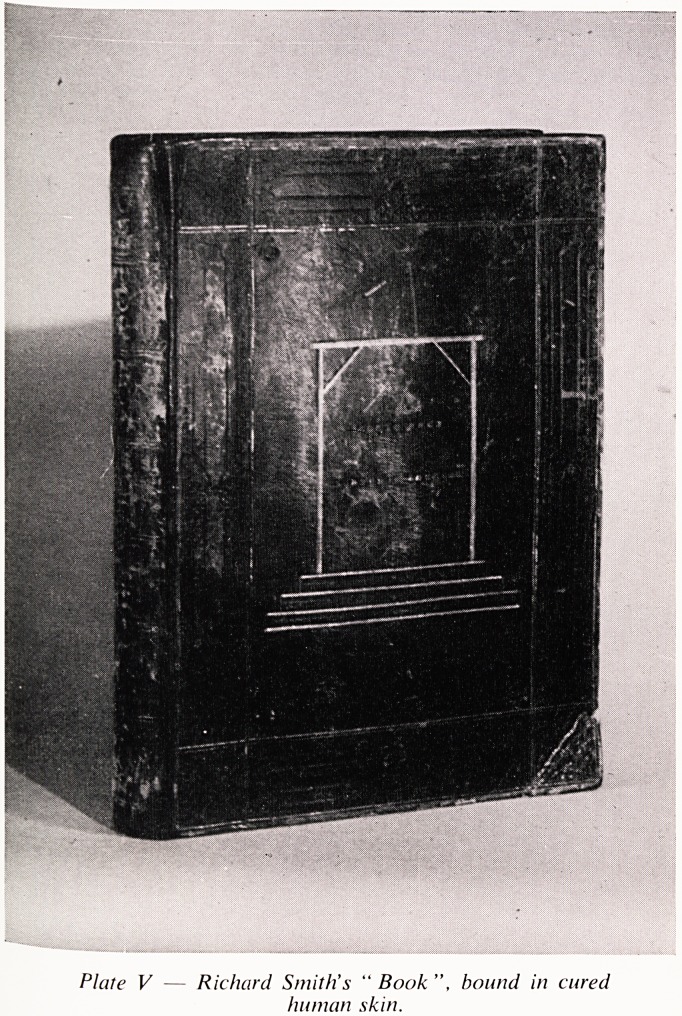# Richard Smith Junior and His Times

**Published:** 1969-01

**Authors:** A. L. Eyre-Brook


### RICHARD SMITH JUNIOR AND HIS TIMES

BY

A. L. EYRE-BROOK

*Presidential Address to the Bristol Medico-chirurgical Society, 9th October, 1968*

The Bristol Royal Infirmary is extremely fortunate in having a wonderful record of its early transactions, in which we can trace the transition from the Julian to the Gregorian Calendar in 1751, when the 11 days were lost, the first of the year moved from the 25th March to the 1st January and the end of the financial year moved from the 24th March forward by 11 days to the 4th April, where it has remained to dog our existence ever since. The records of the Out-patient and In-patients show a fascinating spectrum of diseases which, despite their poverty in science and the relative impotence of medicines or surgery in those days, claim as good a cure rate as do the contemporary enthusiasts, and no doubt for the very same reasons.

Many of you will have read a "History of the Bristol Royal Infirmary" by Munro Smith, first published in 1917, and will know the story of the discovery of all these valuable documents by Richard Smith junior in the year 1791 while he was a medical student at the Infirmary. In his own words "At the time I observed in the hands of a nurse a parcel of papers intended for the common uses of the ward and I was rather surprised to find that they were official letters addressed to the Governors of the Charity. I questioned her as to the means by which she obtained them and was answered very coolly . . . . 'Where we get them all—from the old ward'". I will leave it to you to interpret the meaning of the phrase "intended for the common uses of the ward" and will continue to report that Richard Smith collected many of these documents and with great industry and diligence arranged them in chronological order as the 15 Volumes of Biographical Memoirs, to which he wrote a most interesting introduction in his own fair handwriting. I shall confine myself to references to the times, work, and character of one of the most vivid personalities who strutted on the stage of this Institution—Richard Smith Junior himself.

But I must start with a reference to the older Richard Smiths, for his father and grandfather were also so named. The grandfather was a native of Warminster, and he came to Bristol and established himself as a brewer and maltster in this city. He is reported by Munro Smith as being a man of elegant and refined manners, fond of books and unfitted for his business, which was managed principally by his wife. Richard Smith Senior was one of their eleven children. (Plate I)*. He, the elder surgeon, was born at Counterslip in 1748; at nine he went to the Grammar School in Christmas Street (its fine portico is well preserved to this day) but later to the Grammar School at Warminster, where the discipline appears to have been too severe for his taste, and Richard ran away. Later he went to Winchester College and in 1762 was apprenticed to John Townsend, Surgeon to the Bristol

*I am indebted for this, and for the other photographs, to the Board of Governors of the United Bristol Hospitals.

Infirmary, at the tender age of 14 years. When indentured he convenanted that " Goods he shall not inordinately waste, taverns he shall not frequent, at dice he shall not play, matrimony he shall not contract ". Townsend did his best to keep his apprentice in order but had his difficulties, and after some altercation with the young man about getting home in time, the surgeon would neither see the apprentice nor return any portion of his fees; a lawsuit followed in which Richard pleaded for himself and won a verdict. He then studied in London, and in 1768 returned to Bristol and began to practice; and in that same year he was elected surgeon to St. Peter's Hospital, when he was only 20 years old. On December 6th 1774 he was elected surgeon to the Bristol Infirmary at the age of 26. He was a very successful surgeon and for many years made a large income and would have died a rich man but for his fondness for pleasure and society, and for his friendship with a Mr. John Archer, who followed the rather frivolous and dissipated career of a " blood " in those days. Richard Smith Senior died at the early age of 43, leaving two children, the younger being Richard Smith Junior.

According to Mr. Harsant in his Presidential Address to this Society in 1899, the elder surgeon, Richard Smith, seems to have been a man of great personality, ability, and industry and bequeathed to his son a museum containing nearly a thousand specimens; so the museum originated with Richard Smith Senior. Mr. Hetling reports that Everard Home came down to Bristol from London to view the museum collected by Richard Smith Senior while he, Hetling, was a student. He reports that " after going over every individual specimen which occupied several hours ", Home declared that " it was the most unique, skilful and extensive museum he had inspected as the production of one individual except of course the unrivalled Hunterian Collection ".

Richard Smith Junior, as we shall later see, inherited the same genius for industry and the collection of specimens. This must have started early, as we are told that his own milk teeth were carefully collected as they were shed and fastened on a card to form one of the curiosities in this collection. His passion for collecting veered towards the morbid specimens, especially anything gruesome or uncanny, as we shall see in referring to the trial and execution of John Horwood.

Richard Smith Junior was born in Queen Square on the 28th June 1772. He was undoubtedly a very vivid character, of ruddy complexion with large whiskers and a loud strident laugh which accompanied or followed his own stories or remarks and those of his friends (Plate II). He was described by Mr. Paul Bush as a man of great virility and strength, fond of society and good wine, a great freemason, ready to quarrel but full of pluck and life, and ever ready to stand up for his colleagues. He became indentured to his father as a medical student and when his father died in 1791 he transferred his indentures to Mr. Godfrey Lowe, Senior Surgeon of the establishment and one of the great surgeons of its history. He was elected Surgeon to the Bristol Infirmary on the 23rd June 1796 at the age of 24, became Senior Surgeon in 1812 at the age of 40, and remained on the staff of the Infirmary until his death on the 24th January 1843 at the age of 70. At that age he was really quite incompetent, and this led the Board to enact a regulation shortly after his death stating that surgeons should serve the Bristol Infirmary for no more than 20 years. He was a member of the Town Council in Bristol for a number of years, a Charity Trustee, and at the time of his death Deputy Provincial Grand Master of the masonic lodges in the Bristol district. He was fond of a song and sang lustily. His death was marked by a round composed in his memory:

> " He is gone a fine old fellow;
> For others, his heart was mellow,
> In life all men his friendship found,
> Let now his memory circle round ".

He was undoubtedly a man of literary taste, a facile writer and a born biographer. Munro Smith goes as far as to write in the preface to his admirable book that " Richard Smith not only put in his notes facts and dates but he describes minute details of dress, manner and appearance in a way that has perhaps never been excelled except by James Boswell ".

Richard Smith took part in the controversy as to the date of the foundation of the Bristol Infirmary and its claim to being the first hospital for the sick poor supported by the benevolence of the citizens, to be found in the provinces or Scotland. There was undoubtedly some confusion about the date, and Munro Smith found for instance that the date of foundation printed on the front of the Infirmary had been twice altered by order of the Committee. In one of the minute books there is the following entry: " August 6th, 1828. Mr. Richard Smith having intimated that he had no previous documents relating to the Institution, resolved that the date of the Inscription on the front of the building be 1736 ", and in this he would appear to have been completely accurate. The Bristol Infirmary was, of course, completely rebuilt between 1784 and 1810 and its front was then made to face north with a rear entrance in Earl Street, which street no longer exists. At this date its complement of beds was 180. It was not until 1850 that the Infirmary became the Bristol Royal Infirmary by Royal decree, when the Whig-sponsored General Hospital was already in being. Mr. G. E. Saunders then made the now famous remark: " The patients who want a sovereign remedy will now go to the Bristol Royal Infirmary but those who want a radical cure will go to the Hospital ".

Most of the general practice in the city was then done by apothecaries, who were a most important body of men. They dispensed their own medicines and many of them in addition kept open shops for the sale of drugs. The apothecaries flourished in the 18th century but the era of the physician and the dispensing druggist was already upon us by the turn of the century. On the foundation of the Infirmary the resident apothecary was appointed to do dispensing and to live on the premises and in as much as he was the only resident officer, he must have acted in addition as a kind of house surgeon. He was to be an unmarried man but it seems to have been an appointment much sought after, and it evidently was a position of considerable importance. At many of the large London hospitals the office of apothecary continued at least until the middle of the 19th century and the duties of the office are described as " very varied and dignified " (Harsant). Prior to 1803, Richard Smith Junior lived at 17 College Street where was displayed " Richard Smith, Surgeon ", but on the back door in Lamb Street we find " Richard Smith, Surgeon and Apothecary ".

Problems of canvassing for appointments to the Bristol Infirmary and St. Peter's Hospital were great as competition was very brisk, and it was thought to be of great advantage to get in early. This led to what some thought was impropriety and gave rise to much ill-feeling. There were 20 surgeons in Bristol in 1793 and no more than 9 or 10 appointments at these institutions, and that such appointments were calculated to confer honour upon any professional gentleman was admitted on all hands.

In 1807 there was much debate at the Bristol Infirmary about a ruling to run as follows: " That every election of a Physician or Surgeon to this Charity shall be for the term of 10 years and that the Physician or Surgeon whose term shall have expired by efflux of time may be an eligible candidate for any subsequent vacancy except that which the expiration of his own term shall occasion ". The new rule met the approbation of the Board but had to be laid before the Subscribers, and at this time Richard Lowe was applying for election as surgeon. The rule was being enthusiastically advocated by a group to which Richard Lowe referred in a letter in the following terms . . . . " The Friends of the Charity as they thought proper to style themselves took good care to enforce their argument by bringing numerical force to back them ". But the Trustees were not agreeable to the order of the official Board, and the hopes of the Friends of the Charity were not realised. They began to see that the plan was not exactly what it had been represented, and that it was the last hope of a party to introduce to the " House " physicians and surgeons who had little or no chance in the ordinary way. Those opposing the measure alleged that the rapid succession would introduce half-educated men into the " House ", the effects of which would be truly deplorable. Someone instanced a case where a practitioner, unaccustomed to operations, having imprudently attempted the removal of a cancerous breast, fainted in the midst of the operation, and the patient's life had nearly been sacrificed. It was contended that time alone gave a facility, a steadiness, and a correct judgment to a surgeon and that " the present rule would multiply the list of deaths, which was at present very small ".

We may recall that after Richard Smith died in 1843 at the age of 70, having remained on the staff of the Infirmary until that age and for a period of 47 years, the Board did enact a regulation stating that surgeons should serve the Bristol Infirmary for no more than 20 years.

The physicians and surgeons were as a rule very attentive to their duties, as is seen in a short note from Richard Lowe to Richard Smith preserved in the Memoirs: " My dear Smith, can you take care of my casualties for me from 6.0 till 8.0 this evening as I am obliged to go a short distance out of Town ", signed " Richard Lowe ". However, there were physicians and surgeons who took these duties too lightly and there are numerous references to the Visitors having to expostulate with certain members of the staff who were irregular in their attendance at the Infirmary and it was no doubt from Visitors faced with such duties that the Friends of the Charity were most readily recruited.

I should now like to quote fairly extensively from the record by Richard Smith of the events which led up to the trial and execution of John Horwood for the murder of Hannah Elizabeth Balsam, as it throws so much light on the contemporary scene as well as upon the character of Richard Smith himself.

The events took place in 1821. The record is headed " A few particulars respecting the murder as far as I was personally concerned—Richard Smith ".

*Plate I — Richard Smith, Senior.*
*Portrait in the Board Room, Royal Infirmary*

*Plate II — Richard Smith, Junior.*
*Portrait in the Board Room, Royal Infirmary*

and the story continues: "Coming into the Out-patient waiting room I saw a young woman with a bandage upon her head and enquired what the case was. Was informed that she had been struck by a stone, that she had been there the day before. She had walked from Kingswood without any inconvenience, she had been desired to stay before but she would not. Being too well aware of the insidious nature of such injuries and how readily the apparently most trifling accidents terminate most seriously and fatally, I took great pains to induce the girl to remain. She felt so little that it was with the greatest difficulty that she yielded up the point".

The case apparently progressed well for some days, then the wound became inflamed and the patient febrile and restless. Richard Smith continues, "I considered it to be my duty to summon an immediate consultation —Messrs. Hetling, Lowe, Daniel and Nathaniel Smith—at the bed. We were unanimously of opinion that some serious mischief had commenced and that an incision should be made with a scalpel to examine the state of the skull. This being done and the outer table of the cranium being depressed, it was agreed to remove that portion with an instrument called a trephine. This cut out a circular piece of the size of a shilling and upon this part the stone had fallen (Plate III). The dura mater, or thick membrane, of the brain now being exposed, exhibited a decided mark of a severe inflammatory action having made some progress. The girl was much relieved, but nevertheless was in great pain. Being now of opinion that the girl would not recover I thought it proper to go to the Council House and represent the matter to the Magistrates, that they might take what measures they pleased in regard to the man who had inflicted the injury".

The arrest does not appear to have been accomplished without difficulty and in Officer Bull's narrative, Horwood is reported as armed with a hammer but possibly only a toy hammer as alleged by the defence. A loaded pistol referred to in the same narrative was certainly in the hand of his fellow Officer, and one may doubt whether the attempt at rescue was as serious as suggested in this somewhat melodramatic account. Richard Smith continues "I was informed the next day that Horwood was in custody. Hannah Balsam rapidly declined and I found again needful to report her condition to the Council House. Mr. Alderman Haythorne, accompanied by the Clerk of the Magistrates, Mr. Henry Day, went to the Infirmary. The prisoner was also brought in the custody of the two Officers that the girl might identify him . . . . This having been done, he was then removed from the ward to the consultation room where at that period were deposited the skeletons of Bobbitt and Davis, the infanticides, executed in 1802, and I heard that someone opened the doors and showed them to him but it did not happen I was there". This was a very callous action and it is pleasing to note that Richard Smith clearly wishes to be dissociated from it.

These two skeletons survive in the remnants of the museum. They had been handed to Mr. Godfrey Lowe in pursuance of the Act of Parliament, George II, 1752, which reads:—

"Whereas the horrid crime of murder has of late been more frequently perpetuated than formerly . . . (etc.) contrary to the known humanity and genius of the British nation and whereas it is thereby become necessary that some further terror and peculiar mark of infamy be added to the sentence of death. Be it enacted . . . (etc.), that such body shall be delivered to be dissected and anatomised, provided that it be not hung in chains, and that in no case whatsoever the body of any murderer shall be suffered to be buried unless after such body shall have been dissected and anatomised in the same manner as is now practised for the most atrocious crimes, and any person or persons forcibly taking away such body from the custody of the Sheriffs, their Deputy or the Surgeons, shall be adjudged guilty of felony ".

Hannah Balsam died on the 17th February, and in the April Assize Horwood was tried for her murder. Richard Smith continues " I had upon decease of the girl removed the skull cap and took it with me to the Court but upon reflection I thought that its production might produce a sensation injurious to the prisoner and therefore I kept it concealed ". In spite of this comment it was the opinion of some that Richard Smith took a marked and unbecoming interest in this trial and this comment in the margin of a brief is quite revealing; it runs: " in confidence this gentleman will be much disappointed if the prisoner is acquitted ".

Horwood was found guilty of murder—death occurring 22 days after the injury. Richard Smith contended in evidence that an abscess of the brain was the cause of death; the skull was injured but not much, there being a small indentation about the size of a finger nail on the outer table of the skull; the indentation therefore would not necessarily press on the brain. The abscess was immediately under the wound. The poultices used on the wound—a matter raised by the defence—were not likely to aggravate the injury. The inner table of the skull was diseased in consequence of the inflammation (Plate IV). The treatment which she received was correct—with the operation of trepanning.

Plate IV confirms Richard Smith's contention that the inner table was diseased in consequence of inflammation. These slides were provided by Dr. Hunt, who observed the absence of inflammatory changes on the piece of bone removed by the trephine. It is possible that some bony excresences were rubbed off this flat piece of bone in the course of handling and storing, and it was of course removed 7 days before death.

The relevant dates are: Injury about 26th January, admission about 29th January, trephining 10th February, death on 17th February nearly 22 days after the injury. The changes seen on the inner table of the skull could not have arisen in the 7 days between trephining and death, but the longer interval between the infliction of the open depressed fracture of the outer table of the skull and the death, 22 days later, would have been an adequate period.

The remainder of this tragic tale gives a vivid picture of some aspects of life in those days. These events occurred in 1821, and in order to get a correct perspective we might note that in 1810 the Archbishop of Canterbury and six bishops voted against a Bill for abolishing the capital punishment in cases of stealing, without violence, goods of less value than five shillings (admittedly, the value of the five shillings was very different in those days). To restore some degree of confidence in our ancestors we must, however, note that there were only 53 people hanged in Bristol between 1741 and 1835 a period of 94 years. The hanging before that of Horwood was for forgery (in 1816) and two hangings intervened between Horwood's in 1821

and 1832, when four men were hanged for rioting in the famous Bristol Riots of that year. So that in spite of the riots there were only eight deaths by hanging in 17 years.

Referring to the execution, Mr. Smith's record continues and is worth quoting in full:

"In the morning of the execution I was invited to breakfast with one of the Sheriffs, Robert Jennings Esquire, who resided at the end house of Redcliffe Parade and upon arrival of Mr. William Hare the under-Sheriff, we went to the gaol and were shown into the parlour of Mr. Humphries. There were about 16 persons there, chiefly well dressed females. Shortly after, Horwood came in attended by half a dozen constables. He bowed awkwardly, seemed to be suffering great mental agony, looked around and said "Pray for me, do pray for me". This produced a sort of stifled shriek of horror amongst the assemblage. They knelt down one after another and presently one female began to pray aloud and by her manner and easy flow of words, I had no doubt that she had been accustomed to address an audience extemporaneously. Horwood remained standing but listening with great attention and evidently accompanying the speaker mentally but he was perfectly silent. This lasted about ten minutes when the female, who seemed to be rather above the ordinary class but not a gentlewoman, ended and arose. Horwood then walked round the room and shook hands with all who presented themselves. I was not amongst the number for obvious reasons; in fact, I stood behind a person lest he might recognise me and that my having given evidence against him and even my errand might flash across his mind. He now wrung his hands bitterly, seemed in great distress and exclaimed "O Lord, O Lord". The officers then stepped forward and bound him and speedily left the room. The Reverend Mr. Day then walked before him, reading the burial service. Almost everyone was greatly affected, many shed tears and I believe that I did not escape the contagion. The funeral service of the Church of England is at all times affecting and under these circumstances its effect is irresistable to those who have any feeling. I certainly felt at the moment an indescribable sensation of depression and lowness of spirit. I now went up to the opposite leads which looked down close upon the scaffold where the culprit was just arrived but there was a great bustle and the impression was that there had been some resistance or attempt at escape but we soon learned the cause. The fact was that the heart of the executioner failed him and he slipped away and hid himself as soon as he observed the near approach of the criminal. After some search he was found behind a door and brought up to the scaffold. Horwood behaved very well. He appeared to be absorbed in prayer. The rope was now adjusted and the people began to leave the platform and my courage, if courage it is to be called, failed me. I perceived that the fatal moment was approaching. I was unable to look any longer upon the criminal; I drew back almost involuntarily, turned my face from the scaffold. In a few seconds I walked towards the stairs and bent my steps down them and then towards Mr. Humphries' parlour where I found a few of the females, who I had left there, as also Mr. Sheriff Jenkins. In about ten minutes Mr. Hare, the under-Sheriff came in, bowed to the Sheriff and notified to him officially that the criminal had suffered the sentence of the law. Soon after this Mr. Humphries came in and advised me to quit the gaol at once, intimating that it would be impossible to do anything in regard to the body that evening and perhaps even the following day, at all events not before it. We had good reason afterwards to know that this was pruden advice for it turned out that Horwood's friends, aided by a large body o colliers and stone quarriers, had laid a plan for rescuing the body by rushin sudden upon the escort; while some were fighting, others would have carrie it to a boat ready for the conveyance and it would have been rowed up t Hanham and recovery out of all possibility. The men lay in waiting all th evening and night and, being not aware of the removal, came for the sam purpose the next night. On the Saturday, being the day after the executio I asked a coachman whom I knew if he had any objection to go with me t the gaol to fetch a parcel. He answered " O No Sir, I know what you mean " I stepped into the coach and we drove to the gaol. We were let into th Court, the body was in a room under the drop and perfectly naked. I gath ered up the ropes and cap (no doubt for his Museum). Mr. Humphries the sent for some men and ordered them to put the body into the coach but the one and all most peremptorily refused to go near it. Finding that his authorit went for nothing, I partook myself to an argument which was irresistable. showed two of the fellows a half-crown and assured them that it would b theirs when Horwood was in the coach. One of them said to the other " Com Tom, what dost say? Come, lay hold of him ". This was done. I wrappe around the upper part of the body an old Irish cloak that it might not b seen through the windows and it was pushed into the corner. I was upon th point of closing the door when David Morgan, one of the Sheriff's yeoma said, " Sir would you like me to accompany you?" This opened my eyes t the awkward predicament in which I should find myself if by any acciden the contents of the coach should be discovered during the transit. I therefor gladly accepted his offer. All being ready and no-one allowed to leave th prison but ourselves, the doors were opened and we drove off with as muc speed as our cattle would allow. We passed the New Bridge, through Prince Street, went up Marsh Street, crossed St. Stephen's Street, went into Christma Street and through Lewins Mead to Earls Street, where there was situate the lower door of the Infirmary. We met with not the slightest interruptio Upon our arrival I jumped out and called to some persons belonging to th Infirmary. The body was borne out of the vehicle, at this instance passed soldier and a woman, both of whom appeared astonished but passed on. discharged the coach and the whole affair was fortunately accomplished. Th body was placed upon a trepple in the dead house. When this matter wa all at an end and I was walking quietly home, I confessed that I began t feel very conscious that I had acted rashly and unnecessarily exposed myse to danger and ridicule in passing so encumbered through some of the ver lowest streets of the city, where the least suspicion would have immediatel aroused the population. Many of the multitude who went to see the executio did not look upon Horwood in the light of a common murderer and cried ou against " The body being cut up by the doctors ". In fact, I felt very thankfu that all was quiet. Had I reflected at all, I should have stipulated for deliver of the body, instead of which I gave to Mr. Hare, the under-Sheriff, receipt for the body before I left the gaol on the day of the execution an thus took the thorn out of his foot and put it into my own. On the Sunda Mr. Swain took a plaster mould of the whole head and Mr. Frederic Grainger another of the face. Both are in my museum at the Infirmary; th latter is fixed to a piece of board. Mr. George Cumberland of Wells Stree then made a drawing of the body as it lay upon the trepple and Mr. Mintern

*Plate III — Hannah Balsam's skull, with trephine hole, external view.*

*Plate IV — Hannah Balsam's skull, inner table. Note the intact bone of the trephined disc, and the periosteal reaction surrounding the hole.*

a very clever and rising artist, another. He took also a portrait of the prisoner as he stood at the Bar; both are at the beginning of this book ".

"On the Monday the body was taken to the operation room and placed upon the table" (presumably Greig Smith operating theatre). "About 80 persons were present, some being refused who made application. I then delivered a lecture adapted to a mixed audience upon the general structure of the human body and its physiology, pointing out the great and infinite wisdom and power which they exemplified, illustrating the whole by such drawings and preparations as bore upon the several subjects touched upon. The next day there was another full audience and to them I demonstrated the contents of the chest and lower belly, detailing in a popular way the uses and various functions of the parts, as before, rendered as intelligible as possible by the help of the museum and portfolio. After this, the large muscles were demonstrated in two lectures, after which I notified that the public delivery was at an end. The body was then removed to the dissecting room adjoining the dead house, where the students of the house went on with the dissection until the bones only remained and the skeleton was then disjointed and put into water for maceration. At the same time the skin was undergoing the process of tanning in the adjoining tub. I received the material and instructions for the process from the Sheriff's boot tanners. The skin was also dressed at Bedminster previously to being sent to Essex for the purpose of forming the covering of this book ".

"This book" refers to the bound volume of all the papers connected with this case and embossed on both front and back are the words "*in cutis vera*" —an example of a somewhat unpleasant trait in Richard Smith's character (Plate V). The volume also contains a phrenograph of John Horwood, an example of the pseudo-science of those days which really demanded little more from a credulous public than the "Black Box" and other such artifices of our present time. Neither Mrs. Schimmelpennick, who constructs the phrenograph, nor Mr. Spurzheim the originator of the phrenological theories for the diagnosis of character (who was at the time living in Bristol), could find the "bump of murder" well developed. They contended that the chief mental characteristics were "combativeness", "self-esteem", and "hope".

Thus we conclude this distressing narrative of an injury so slight, the application of poultices, major surgery under such septic conditions late in the illness, and a fatal termination. Horwood's offence against society would appear to have been no greater than that of others who were sentenced to death at that Assize in 1821, but he has become a *cause célèbre*. The character of Richard Smith Junior was certainly a casualty in this narrative. But we must not forget that we live 150 years later; in those days, surgeons must have needed to be of very tough fibre.

I now wish to record events leading up to the birth of the Bristol Medical Library, which has had some deserved fame through the years; and to the acquisition by the Bristol Infirmary of the Richard Smith Museum, which was once famous but which has suffered the depredations which time so effectively wreaks on such objects as constituted a museum of those days. In contrast, the John Hunter Museum at the Royal College of Surgeons, which was contemporary, still exists despite enemy action, and Bristol has room for self-criticism in this direction.

A letter from Richard Smith to the Treasurer of the Bristol Infirmary makes admirable reference to the advantages accruing to the surgeon and the patient, to the benefits for the teaching of the junior pupils and to the promotion of research which these additional facilities would foster. The letter is dated the 20th November, 1826:

" Mr. Treasurer, When I have had an operation to perform out of the ordinary routine, or have attended consultations upon obscure and doubtful cases of injury or disease, I have not unfrequently wished that I had the means of immediate reference to anatomical or pathological preparations which might, in the latter instance, have assisted my judgment and in the former, enabled me to act with increased confidence and decision. Cases also have come before me in the exercise of my ordinary duties which have made me regret that the House was not in possession of a medical library. I need not, Sir, I am sure, point out to you the advantage which might have been the result, to the patient, since in matters of moment, the mind and the memory often require to be strengthened and refreshed and, indeed, even with all these aids where the life or limb of a human being is at stake, the personal responsibilities are in my view sufficiently formidable. I have also sometimes lamented that I had not the present means of demonstrating the rationale of an intended operation to the junior pupils, since an exposition of the dangers which surround the hand of an operator would, by creating an anxiety for the safety of the patient, not only insure an immediate propriety of conduct but properly produce a subsequent desire for professional research, so much to be wished for in the student.

These considerations, combined with a general wish to benefit the Charity to the extent of my abilities have determined me to place at the Infirmary my museum and medical books if the gentlemen of the Committee will appoint a suitable apartment for their reception. I have reason to think that upon this point the House Visitors will find no difficulty. I do not give the preparations and books but deposit them only because I consider that as long as I have the honour to be one of your Officers, such an arrangement will be quite as beneficial to all who may be connected with the establishment and more congenial to my own feelings. When I inform you, Sir, that the Museum owed its foundations and a great progress to the labours of my late universally respected father and that I have zealously endeavoured to preserve and augment it during the period of 35 years, I trust that you will excuse the wish to continue for the present its guardian ". He concludes, " I have the honour to remain, Mr. Treasurer, with the greatest respect, very truly, your obliged Servant, Richard Smith ".

The Library, with that of this Society, eventually formed the Medical Library of the University of Bristol, and the important literary works which the Librarian treasures and often displays at these meetings, no doubt largely derive from these early contributions to the Bristol Infirmary Library.

My remarks have covered something of what we know of Richard Smith Junior. It is not enough; a biographer leaves only indirect evidence of himself, and it is of course from Richard Smith and his 15 Biographical Memoirs that we know so much of others who have played their various parts in the history of the Bristol Infirmary. I have given to some merely a recollection of what they already knew, and to others additions to their knowledge, but

*Plate V — Richard Smith's " Book ", bound in cured human skin.*

I trust to all some stimulus to further probings into the rich store of local history with which Bristol is blessed. I cannot conclude without mentioning my debt of gratitude to Richard Smith himself, from whom I have quoted extensively, to Munro Smith and his History of the Bristol Royal Infirmary, to Latymer and his Annals of Bristol and to a former President, Mr. William Harsant, who spoke on a kindred subject.

## Figures and Tables

**Plate I f1:**
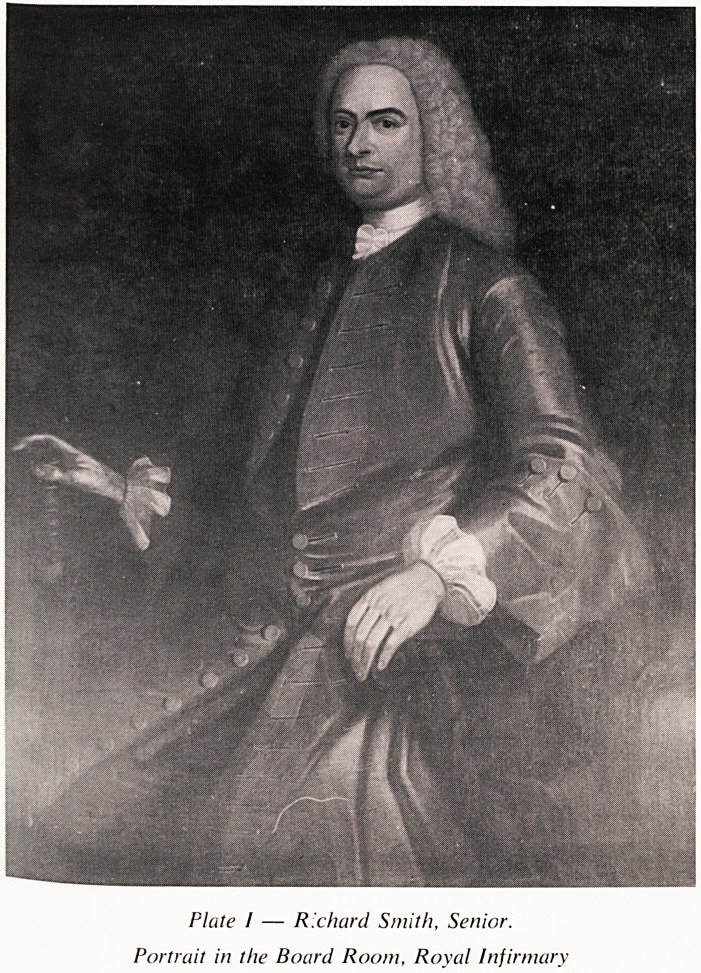


**Plate II f2:**
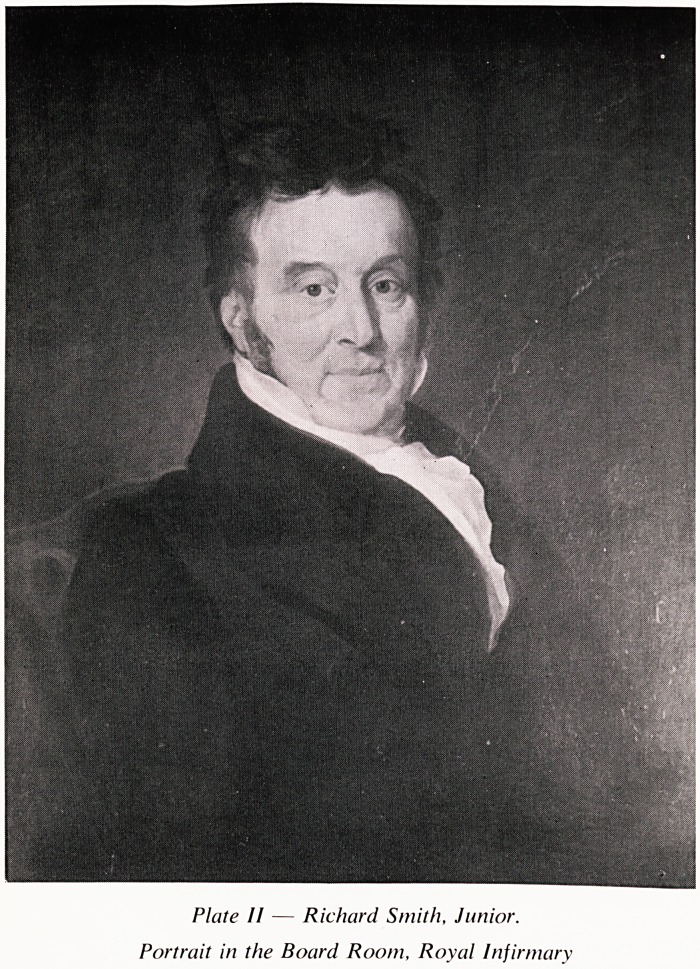


**Plate III f3:**
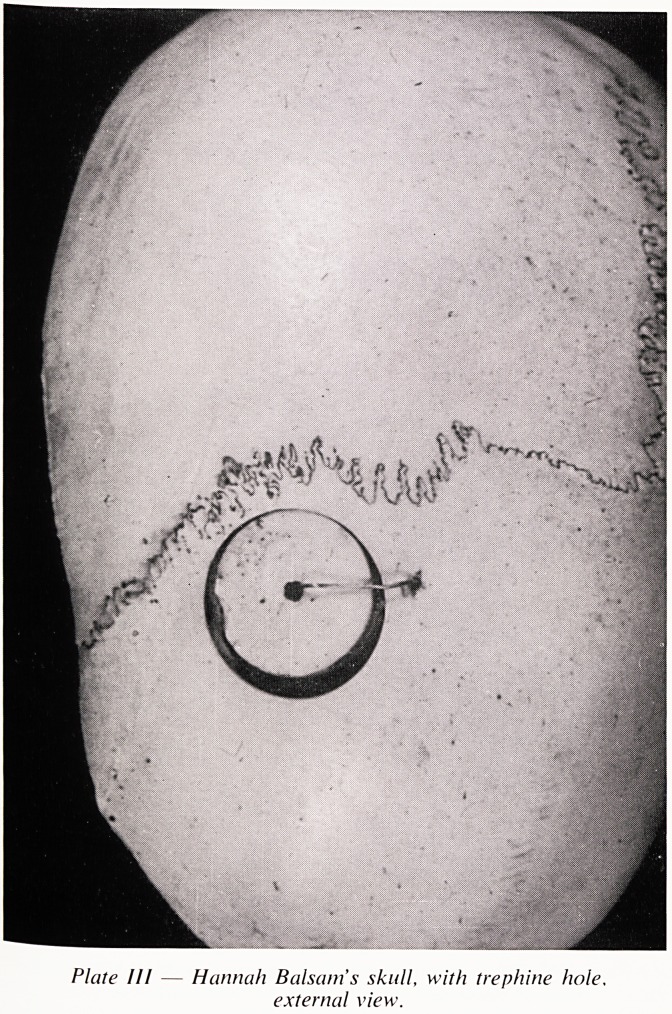


**Plate IV f4:**
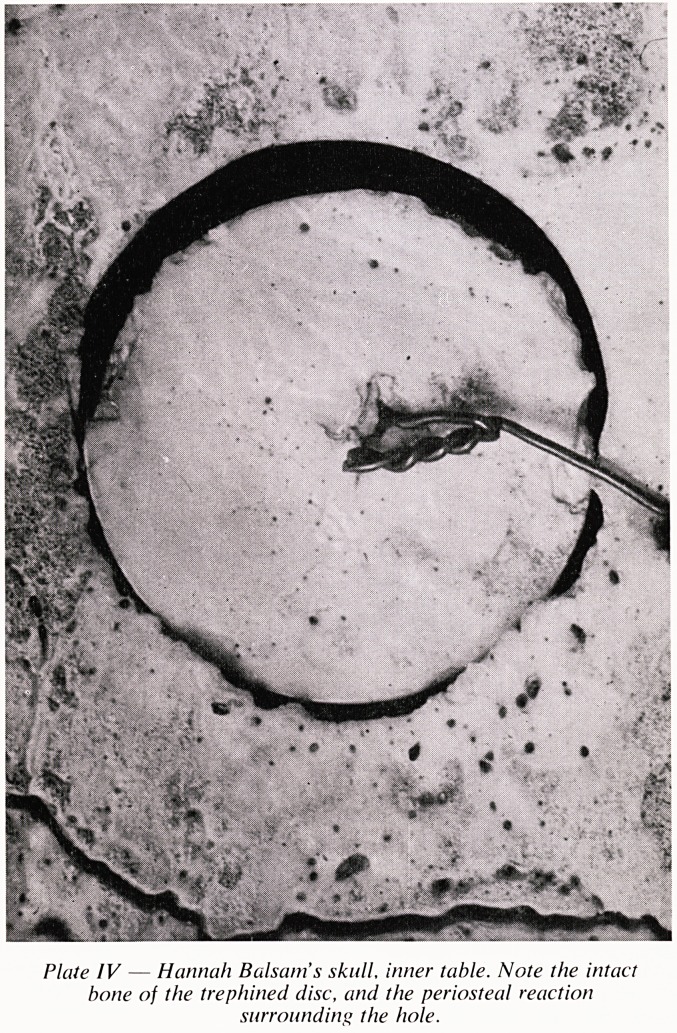


**Plate V f5:**